# Economic consequences of the vaccination against hepatitis A in the Bulgarian healthcare setting

**DOI:** 10.1080/13102818.2014.909654

**Published:** 2014-07-10

**Authors:** Maria Dimitrova, Guenka Petrova, Konstantin Tachkov, Maria Krasteva Bozhkova, Maria Kamusheva, Konstantin Mitov

**Affiliations:** ^a^ Organization and Economy of Pharmacy, Faculty of Pharmacy, Medical University of Sofia, Sofia, Bulgaria; ^b^ Medicine College, Medical University of Plovdiv, Plovdiv, Bulgaria

**Keywords:** HAV vaccine, cost–benefit analysis, cost of vaccination, prevalence

## Abstract

The purpose of the present analysis is to calculate and compare the costs and results of the implication of the inactivated vaccine against hepatitis A virus (HAV) in the Bulgarian healthcare setting in the period of 2002–2012. A combined pharmacoeconomic and epidemiological study was performed on the basis of the prevalence of hepatitis A infection in this 10-year period. The investments in the vaccination were considered as costs and the avoided costs in the case of vaccination of all one-year old children in the population – as benefits. The results show that the vaccination of one-year-old children would be cost effective to the healthcare system in the years with an epidemiologic outbreak, as in these years the total cost of treatment of patients with hepatitis A infection exceeds the cost for vaccination of the whole one-year-old cohort. The critical threshold is 4600 infected patients per year that equalize the benefits to costs. The inclusion of HAV vaccine in the National Immunization Calendar would be cost effective for the healthcare system when the vaccination is performed in certain risk groups and could help to decrease the circulation of the virus in the general population.

## Introduction

Prophylactic vaccination programmes provide economic and social benefits to the society (increased quality of life, decrease in the productivity losses due to work absence, etc.) as well as to the healthcare system (decreased hospitalizations, treatment of complications, decrease in the morbidity, etc.).[1–6] The economic benefits of hepatitis B and C vaccination have been studied in a variety of settings and health care systems.[7–12] These studies showed that the main economic benefits from the vaccination against hepatitis B and C are the decrease of the chronic and life-threatening complications, which leads to significant health care cost savings and quality of life improvement.[7–10]

Hepatitis A infection is self-limiting and does not lead to chronic complications but in some cases it might be very severe and cause a significant number of deaths.[2,5,12–18] Vaccination against hepatitis A has been evaluated as cost effective in some countries with high risks of infection, but not in others.[4,6,19–24] Therefore, studying the economic impact of the vaccination against hepatitis A is important to define its healthcare and social reasoning.

The aim of this analysis is to calculate and compare the costs and results of the implication of inactivated vaccine against hepatitis A virus (HAV).

The point of view of the analysis is that of the healthcare system and the time horizon is set on the basis of the clinical trials data for the duration of protection of vaccination – 10 years.

## Methods

A combined pharmacoeconomic and epidemiological analysis was performed on the prevalence of hepatitis A for a 10-year period (2002–2012). The investments in the vaccination were considered as costs and the avoided health care costs in the case of vaccination of all one-year-old children in the population, as benefits.

### Epidemiology analysis

The data used in the epidemiology analysis were collected retrospectively from the registry of the National Center of Public Health (NCPH) for the number of registered hepatitis A cases in the observed 10-year period (2002–2012).[25]

### Cost analysis

The considered direct health care costs include hospitalization, ambulatory pharmacotherapy and monitoring tests of the cohort of infected patients for the given year.[26,27] Hospitalization costs and monitoring laboratory tests were taken from the National Health Insurance Fund (NHIF) tariff.[28] The number of hospitalized persons was multiplied by the cost of hospitalization according to the NHIF tariff. Pharmacotherapy costs for ambulatory patients after hospital discharge were calculated on the basis of previous analyses for the standard therapeutic schemes used.[9,29]

The following formula was used to calculate the total medical costs:
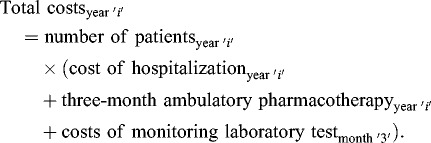



To calculate the vaccination for each cohort of one-year-old children, the number of children was multiplied by the price of the vaccination regime (two vaccine doses) in the given year. The data for the number of newborns was taken from the National Statistical Institute.[26] The vaccine considered in the analysis is that available for the whole observed period and the one with the lower price. The price of the vaccine was taken from the Positive Drug List (PDL) Register.[27]

### Cost–benefit analysis

In the cost**–**benefit analysis, the total medical cost for each year was subtracted from the cost of vaccination for the same year. To evaluate the profitability of the vaccination programme, the difference was illustrated graphically for each year and totally for the whole period.

The net benefit threshold was calculated by using the following formula:[30]
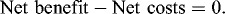



To equalize the net benefit, the average number of infected patients was varied with a 100-step within the interval of ± 30%.

### Sensitivity analysis and statistical methods

Sensitivity analysis was performed to evaluate the effect of the uncertain input data in the model: number of patients, cost of clinical pathway, ambulatory pharmacotherapy costs, cost of laboratory monitoring tests at third month, cost of vaccination and number of one-year-old children. We assumed that these parameters could vary through the period and to have impact on the net-benefit result.

The uncertain input data were varied in the interval of ±30%. A tornado graphic was applied to show the impact of the varied input parameters on the net benefit.

A statistical analysis with Mann–Whitney test was performed to evaluate the statistical difference between the number of patients in the years of outbreaks with that in the rest of the period and the costs of treatment in the outbreak years with the cost of treatment in the rest of the years.

All costs are presented in national currency Bulgarian leva (BGN) at the exchange rate of €1 = 1.95 BGN.

## Results and discussion

The present analysis surveys a situation with real prevalence data and actually spent health care and financial resources for the treatment of patients with HAV infection and draws a comparison with the hypothetical costs on probable vaccination of the whole cohort of one-year-old children.

The average number of registered patients with hepatitis A for the observed 10-year period is 3738 (Table 1). Two epidemiological outbreaks were registered in 2005–2006 and 2011–2012, respectively, with the number of registered patients reaching highest levels of 7266 in 2006 and 5588 in 2012 (Table 1). The morbidity in 2003 and 2008–2009 was lower than the average for the observed period. The number of children at the age of 1 is almost constant each year and the average number is 72486 (Table 1).

**Table 1.** Number of reported hepatitis A infections for a 10-year period and number of children aged 1 for the observation period.

Year	2002	2003	2004	2005	2006	2007	2008	2009	2010	2011	2012	Average number
Registered cases	4753	2155	3990	5225	7266	2800	908	1064	2350	5588	5023	3738
One-year old children cohort	68180	66499	67359	69886	71075	73978	75349	77712	80956	75513	70846	72,486

Data from the NCPH show that in the years with epidemiologic outbreaks, especially 2011 and 2012, almost half of the registered patients were children and the predominant morbidity were in the group of 4- to 9-year olds.

The input parameters included in the analysis are shown in Table 2.

**Table 2.** Input model parameters.

Cost of treatment of registered acute hepatitis A infection		
Parameters		Source
Cohort	Registered patients with viral hepatitis A for the period 2002–2012	National Center of Public Health
Hospitalization cost	Increases from 600 BGN to 1000 BGN	National Framework Contract
Ambulatory pharmacotherapy (hepatoprotectors)	Calculated for three-month period for each of the years in the period observed in current prices	[29]
Biochemical monitoring test	10 BGN	National Framework Contract
Assumed costs for vaccination at the National Health Insurance Fund (NHIF) prices		
Cohort	Number of children at the age of 1 for each of the years in the period 2002–2012	National Statistical Institute
Vaccine–immunization plan (1 + 1)	Inactivated HAV vaccine – 56.64 BGN	Positive Drug List (PDL)

**Figure 1.**
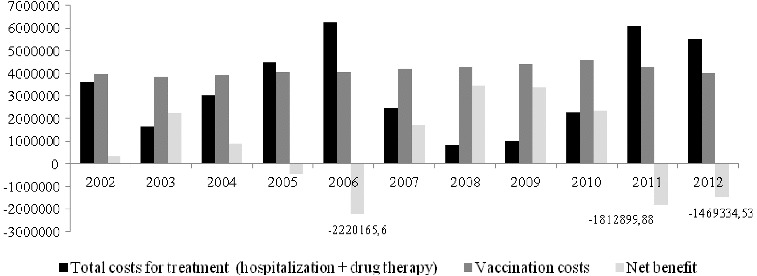
Difference between the total treatment costs (in BGN) and vaccination costs per year.

The mean cost of treatment amounts to 3.3 million BGN annually but in the years with epidemiologic outbreaks there was a twofold increase from the mean value and a fivefold increase from the lowest costs for treatment (Figure 1).

When comparing the treatment costs and the costs of vaccination which the NHIF would have paid for the vaccination of all one-year-old children in the observed period, the result shows that vaccination is cost-effective investment which is paid out in the years with epidemiologic outbreaks (Figure 1). This means that the treatment costs of all registered patients with hepatitis A were higher than the costs that would have been paid for the vaccination of all one-year-old children (100%) in the same year (Figure 1). In these years, if vaccination had been carried out, the healthcare system would have saved from 1.5 to 2.2 million BGN from hospitalizations and additional pharmacotherapy costs.

**Figure 2.**
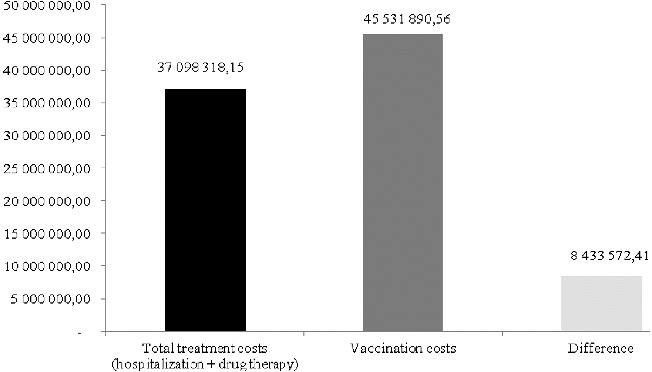
Aggregate net benefit for the period 2002–2012 (in BGN).

The aggregate comparison of the treatment costs for all patients in the period 2002–2012 and the vaccination costs which NHIF would have paid for all one-year-old children in the same period shows that the total costs for treatment that NHIF has already paid are 37 million BGN and the costs for vaccination would have amounted to 45.5 million BGN on the basis of the current vaccine price (Figure 2). The additional vaccination costs that the society would pay amount to 8433572 BGN.

**Figure 3.**
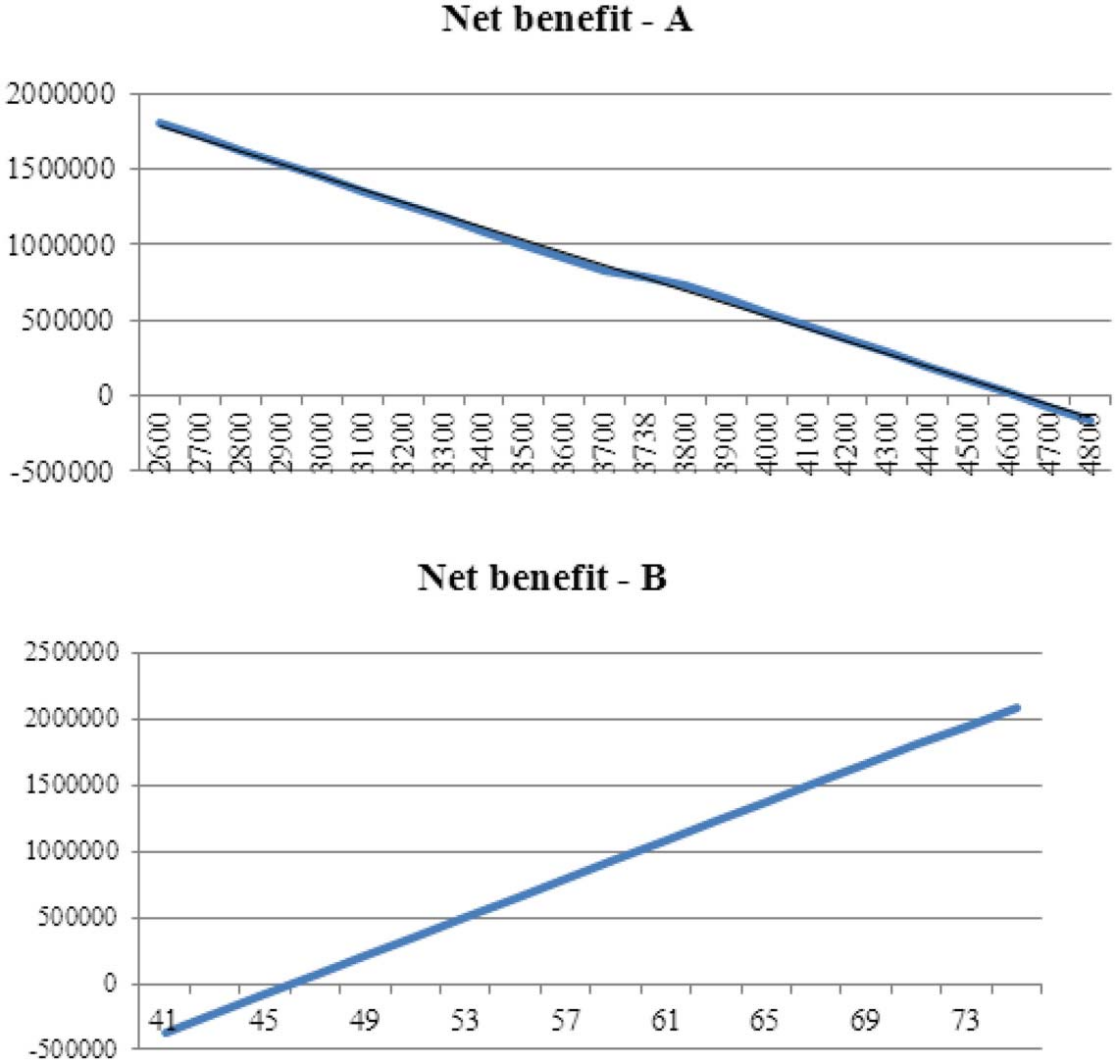
Impact of the variation of number of patients with HAV infection (A) and the variation of cost of HAV vaccine (B) on the net benefit.

The variation in the average number of patients with HAV infection with a 100-step in the interval of ± 30% (2600–4800) shows that the vaccination programme would be beneficial for the healthcare system if the morbidity is more than 4600 people per year. The variation in the average cost of vaccination with a two BGN step in the interval of ± 30% shows that the vaccination programme would provide benefits to the healthcare system if the price of the vaccination scheme is below 45 BGN (Figure 3).

The performed Mann–Whitney test shows that there is a statistical difference between the median number of patients in the years with outbreaks and the median number of patients in the years without an outbreak (5500 vs. 2400; *P* < 0.0001). The same is the influence on the cost of treatment in the years with outbreaks and the cost of treatment in the years without outbreaks (5.6 million vs. 2.5 million; *P* < 0.0001)

**Figure 4.**
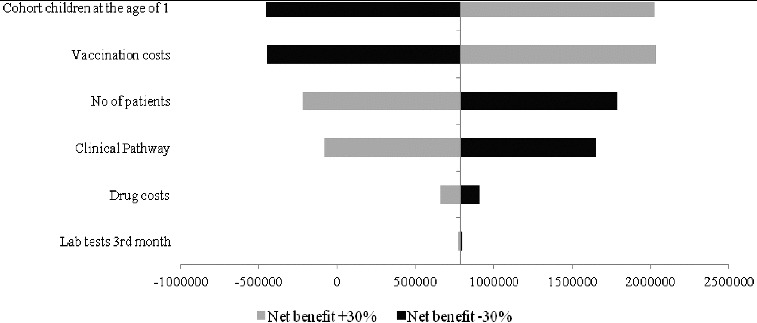
Tornado diagram on the impact of uncertain input parameters in the model on the net benefit.

The results from the sensitivity analysis show that the factors with a major impact on the net benefit would be the variation in the number of patients (morbidity), the vaccination cost and the cost of the clinical pathway (Figure 4).

To our knowledge, this is the first published economic analysis on the economic consequences of vaccination against HAV in Bulgaria. It adds to our earlier overview of the data for efficacy, safety and economic benefits of the HAV vaccines, which did not focus on the situation in the country.[5]

This analysis shows that the vaccination with HAV vaccine would produce savings from hospitalizations and additional treatment when the morbidity in the general population is more than 4600 people annually or if the average price of the vaccination programme is below 45 BGN. It should also be noted that the vaccine price is the registered market price, which would likely be lower in mass immunization because of the negotiation system for vaccine delivery.[27]

Vaccination against HAV infection is recommended worldwide as cost effective for the healthcare system if all one-year-old children who fall in groups with high risk and those living in poor hygiene and sanitation are vaccinated.[4,6,19–24]

According to WHO, 27 countries worldwide, including some European countries, have included HAV vaccination in their national immunization programme in certain groups, e.g. children at the age of 1 belonging to risk groups, healthcare professionals, travellers to highly endemic regions, in terms of outbreaks, etc.[11,31,32]

The results from the present analysis suggest that the vaccination against HAV infection would be cost effective for the Bulgarian healthcare system if applied in certain risk groups. This stems from the fact that in the years with outbreaks the costs for treatment of patients with hepatitis A exceed the vaccination costs of all one-year-old children in the population for these years. Mass vaccination would be profitable for the healthcare system if the morbidity is over 4600 people annually and also would help to decrease the viral circulation in the future, as Bulgaria is a country with intermediate prevalence of anti-HAV antibodies.

Based on the epidemiology review we can assume that the prevalence of HAV in the Bulgarian population is characterized with cyclic epidemiologic outbreaks.[18,25] In this setting, the vaccination would be cost effective for the healthcare system if all risk groups are vaccinated (as laid down in Ordinance 15).[33] This would provide savings to the system from hospitalizations and savings to the society from the necessary ambulatory drug treatment and dietary regime after hospital discharge.

Data from IMS Health Bulgaria confirm that the society indeed needs vaccination against HAV infection, since in the years of outbreaks there is an increase in the HAV vaccine consumption: 1761 in 2006, 1096 in 2011 and 2595 in 2012. Another interesting trend is observed in the years post-outbreak when the vaccination is also high: 1350 in 2007 and 1931 up to September 2013.

At present, the price difference between the two inactivated vaccines for hepatitis A authorized in Bulgaria is negligible and would not change the results from the analysis.[27]

A limitation of our analysis is the fact that we did not consider the mortality in case of severe infection because of lack of data. Availability of such information would make the vaccination more beneficial if the productivity loses due to premature death are added to the avoided costs.

## Conclusions

The results from the performed analysis indicate that inclusion of the HAV vaccine in the National Immunization Calendar would be cost effective for the Bulgarian healthcare system and would help to decrease the virus circulation in the general population. 
